# Antibodies and Vaccines Target RBD of SARS-CoV-2

**DOI:** 10.3389/fmolb.2021.671633

**Published:** 2021-04-22

**Authors:** Long Min, Qiu Sun

**Affiliations:** State Key Laboratory of Biotherapy, Cancer Center, West China Hospital, Sichuan University and Collaborative Innovation Center for Biotherapy, Chengdu, China

**Keywords:** SARS-CoV-2, RBD, antibodies, vaccines, COVID-19

## Abstract

The novel human coronavirus, severe acute respiratory syndrome coronavirus-2 (SARS-CoV-2), which gives rise to the coronavirus disease 2019 (COVID-19), has caused a serious threat to global public health. On March 11, 2020, the WHO had officially announced COVID-19 as a pandemic. Therefore, it is vital to find effective and safe neutralizing antibodies and vaccines for COVID-19. The critical neutralizing domain (CND) that is contained in the receptor-binding domain (RBD) of the spike protein (S protein) could lead to a highly potent neutralizing antibody response as well as the cross-protection of other strains of SARS. By using RBD as an antigen, many neutralizing antibodies are isolated that are essential to the therapeutics of COVID-19. Furthermore, a subunit vaccine, which is based on the RBD, is expected to be safer than others, thus the RBD in the S protein is a more important target for vaccine development. In this review, we focus on neutralizing antibodies that are targeting RBD as well as the vaccine based on RBD under current development.

## Introduction

Until now, three coronaviruses, Middle-East respiratory syndrome coronavirus (MERS-CoV),severe acute respiratory syndrome coronavirus (SARS-CoV), and SARS-CoV-2, have crossed over the barrier and lead to terrible pneumonia in human (Stadler et al., 2003; Mackay and Arden, 2015; Chen B. et al., 2020). SARS-CoV-2 has given rise to a global pandemic because it has more possibility of human-to-human infection than SARS-CoV (He et al., 2020) and MERS-CoV, which results in high numbers of infections and deaths all over the world. Due to the high infection rate, it is an emergency to develop novel vaccines and drugs.

**FIGURE 1:**
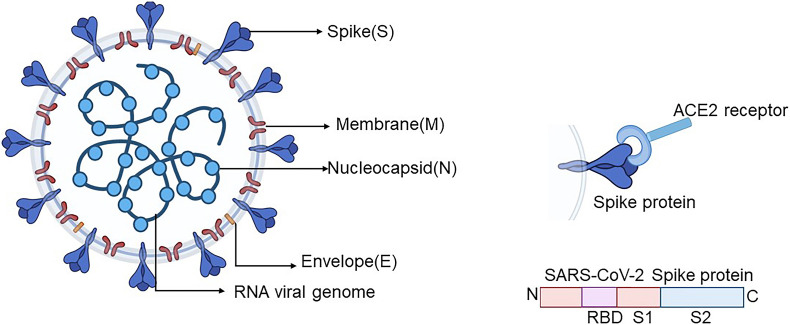
Schematic diagram of the SARS-CoV-2 coronavirus particle. Four structure proteins contain the envelope protein (E), nucleocapsid protein (N), spike protein (S), and membrane protein (M). SARS-CoV-2 virus enters the cell through the S protein on the surface of SARS-CoV-2 by means of binding with its receptor ACE2. The S protein is mainly divided into S1, which contains RBD, and S2 subunits. Created by BioRender.com. ACE2, angiotensin-converting enzyme 2; SARS-CoV-2, severe acute respiratory syndrome coronavirus-2; RBD, receptor-binding domain.

Severe acute respiratory syndrome coronavirus-2 is closely related to bat coronavirus, which generally encodes four structure proteins, namely, spike (S), membrane (M), nucleocapsid (N), and envelope (E) (Figure 1) (Mittal et al., 2020; Wang M.Y. et al., 2020). The entry of the coronavirus into the cell relies on the viral spike protein (S protein) binding to a host cell receptor and human angiotensin-converting enzyme 2 (ACE2), which is primed through the serine protease TMPRSS2. TMPRSS2 entails S protein cleavage at the S1/S2 site with an N-terminal domain (S_*NTD*_) and the receptor-binding domain (RBD, S_*RBD*_) binding to ACE2. The interaction between RBD and ACE2 leads to the conformational change of the C-terminal subunit (S2), which entails the fusion of virus with the cellular membranes (Hoffmann et al., 2020; Walls et al., 2020; Wang M.Y. et al., 2020).

Researchers have put plenty of effort to develop more effective and safer vaccines and drugs against coronavirus disease 2019 (COVID-19), for instance, virus-like particle vaccines, nucleic acid vaccines, subunit and adenovirus-based vector vaccines, neutralizing antibodies, and inactive and live-attenuated vaccines (Lv H. et al., 2020). The traditional neutralization mechanism is to block the receptor-binding site located between the RBD and ACE2. The high similarity of amino acids in RBD between the two viruses can enhance the ability of neutralizing antibodies. Recently, numerous neutralizing antibodies have been found to target the RBDs of MERS-CoV or SARS-CoV (Du et al., 2009, 2017; Chen W.H. et al., 2020). Therefore, searching for neutralizing antibodies that target the RBD of SARS-CoV-2 is essential in the design of the SARS-CoV-2 vaccine.

In this review, we have discussed the recent development of RBD-targeting vaccines and neutralizing antibodies, which have a strong ability to inhibit viral entry through disrupting the binding between RBD to the receptor ACE2, which could provide a wider perspective to the design of useful therapeutic approaches against COVID-19.

## SARS-CoV RBD-Targeting Antibodies With Cross-Reactivity Against SARS-CoV-2

The similar sequence of RBD between SARS-CoV and SARS-CoV-2 is close to 73.5% (Lu et al., 2020), and they share the same receptor ACE2, so it is possible that SARS-CoV RBD-targeting antibodies can exhibit cross-reactivity against SARS-CoV-2. A few antibodies which target SARS-CoV have displayed neutralizing capabilities to SARS-CoV-2 (Table 1) (Ho, 2020).

**TABLE 1:** Severe acute respiratory syndrome coronavirus RBD-targeting monoclonal antibodies with cross-reactivity against SARS-CoV-2.

Ab name	Ab type	Ab source	Neutralizing mechanism
*VHH-72-Fc*	sdAb	Llama	Disrupts the ACE2-RBD interaction (Ho, 2020)
*S309*	lgG	Human	Targets a conserved glycan-containing epitope in S protein (Ho, 2020)
*ADI-55689*	lgG	Human	Binds to the edge of the ACE2 binding site of RBD (Wec et al., 2020)
*ADI-55993*	lgG	Human	Binds to the edge of the ACE2 binding site of RBD (Wec et al., 2020)
*ADI-56000*	lgG	Human	Binds to the edge of the ACE2 binding site of RBD (Wec et al., 2020)
*ADI-55688*	lgG	Human	Binds to the edge of the ACE2 binding site of RBD (Wec et al., 2020)
*ADI-56046*	lgG	Human	Binds to the edge of the ACE2 binding site of RBD (Wec et al., 2020)
*ADI-56010*	lgG	Human	Binds to the edge of the ACE2 binding site of RBD (Wec et al., 2020)
*ADI-55690*	lgG	Human	Binds to the edge of the ACE2 binding site of RBD (Wec et al., 2020)
*ADI-55951*	lgG	Human	Binds to the edge of the ACE2 binding site of RBD (Wec et al., 2020)
*47D11*	lgG	Transgenic H2L2 mice	Binds to the conserved epitope of RBD (Jahanshahlu and Rezaei, 2020)
*7B11*	lgG	Human	Blocks the binding of RBD-ACE2 (Tai et al., 2020)
*18F3*	lgG	Human	Targets an epitope on RBD (Tai et al., 2020)
*H014*	lgG	Human	Competes with ACE2 for binding with RBD (Lv Z. et al., 2020)
*CR3022*	scFv	Human	Destroys the prefusion S trimer (Huo et al., 2020b)

One of the antibodies called nanobody is a novel and unique class of antigen-binding fragments (Fab) with superior characteristics, such as small size, robust antigen-binding affinity, high stability, access to more epitopes, and low production expense, which make it a suitable antibody for the treatment (Jovcevska and Muyldermans, 2020; Koenig et al., 2021). Identifying from a llama that is immunized with COVID-19 spike and MERS-CoV S protein, the nanobody VHH-72, a SARS-CoV RBD-directed single-domain antibody (sdAb), disrupts the interaction between RBD and ACE2 and cross-neutralizes against SARS-CoV-2. Owing to its rapid dissociation, the binding affinity of VHH-72 to SARS-CoV-2 is weaker than that of VHH-72 to SARS-CoV. To overcome this disadvantage, a bivalent SARS VHH-72 (VHH-72-Fc) has been developed, and it exhibits the neutralizing activity with an IC_50_ value of ∼0.2 μg/mL in a SARS-CoV-2 pseudovirus neutralization assay (Wrapp et al., 2020). One antibody named S309 targets a conserved glycan-containing epitope within the S protein on the front end of the RBD in the open and close S state and exhibits cross-reactivity toward SARS-CoV and SARS-CoV-2 (Pinto et al., 2020). In addition, non-monoclonal antibodies (NMAbs) identified through a memory B-cell repertoire from a patient with SARS can cross-neutralize against SARS-CoV-2. Up to now, eight antibodies have been identified to target the RBD of SARS-CoV-2. The median inhibitory concentration (IC_50_s) of the RBD-directed nAbs varies from 0.05 to 1.4 mg/mL, among which ADI-55689 interacts with the fringe place of the ACE2-binding region, which is close to the conservative center domain of the RBD, while ADI-56046 binds to some extent approximately in foreign lands on the flexible tip of the RBD that exhibits neutralizing activity and competes with hACE2 (Wec et al., 2020). 47D11, from immunized transgenic H2L2 mice that are sequentially done with the purified S protein, binds to the conserved region of RBD distant from the spike-receptor interactive binding sites and inhibits the binding of ACE2 and S protein (Jahanshahlu and Rezaei, 2020). Moreover, a few SARS-CoV RBD-based mouse monoclonal antibodies (mAbs), such as 7B11 and 18F3, have shown cross-reactivity against SARS-CoV-2. 7B11 can prevent SARS-CoV or SARS-CoV-2 from binding to ACE2, but 18F3 cannot, which indicates that their epitopes of the receptor-binding site on RBD are different (Tai et al., 2020).

Through phage display technology, a potent humanized NMAb H014 is screened out to target the RBD of SARS-CoV and exhibits cross-neutralizing activity against SARS-CoV-2. Moreover, IgG and Fab fragments of H014 could target the RBD of both SARS-CoV and SARS-CoV-2 to generate neutralization at subnanomolar concentrations with IC_50_ values of 1 and 3 nM against SARS-CoV and SARS-CoV-2 pseudovirus infection, respectively, and with IC_50_ value of 38 nM against authentic SARS-CoV-2 virus (Lv Z. et al., 2020).

CR3022 identified from a SARS survivor exhibits a lower affinity to SARS-CoV-2 than SARS-CoV. The epitope of CR3022 is exposed only when at least two out of the three S^*B*^ domains of a spike-glycoprotein trimer are in the open conformation (Tian et al., 2020; Yuan et al., 2020). Recently, Huo et al. found that CR3022 destroys the perfusion spike trimer with T_1/2_ at room temperature in the absence of ACE2, which is unlike the traditional mechanism of neutralizing coronaviruses by blocking receptor interaction. Furthermore, this leads to a slightly higher neutralization of the antibody, and the (non-inactivated) virus is cleaned off the cells within a short time. In addition, unlike antibodies that compete with ACE2, the ACE2-RBD-binding domain does not overlap with the epitope of CR3022; therefore, it is unusually resistant to virus escape (Huo et al., 2020b). CR3022 has a higher affinity to the SARS-CoV-2 P384A mutant and shows a similar potency of neutralizing activity against SARS-CoV. Conclusively, CR3022 could be used as a potential candidate therapeutic, alone or in combination with other antibodies for the treatment of COVID-19 (Yi et al., 2020).

## Antibodies Exclusively Target SARS-CoV-2 RBD

It is relatively common to find antibodies that have the cross-binding ability to both SARS-CoV-2 and SARS-CoV RBD, but cross-neutralizing responses may be rare (Lv H. et al., 2020). It is essential to design and develop potential antibodies which target SARS-CoV-2 specifically and selectively (Table 2). The human phage display technology is a powerful tool that has revolutionized the development of identifying and optimizing antibodies, providing breakthrough points for further applications in the invention of therapeutic mAbs in anti-infectious diseases (Alfaleh et al., 2020).

**TABLE 2:** Potential antibodies exclusively target SARS-CoV-2.

Ab name	Ab type	Ab source	Neutralizing mechanism
*3F11*	sdAb	Human	Competes with ACE2 for binding to RBD (Yu et al., 2020)
*BD-368-2*	lgG	Human	Disturbs the RBD-ACE2 interaction (Cao et al., 2020)
*Ab1 lgG1*	lgG	Human	Disturbs the RBD-ACE2 interaction (Li W. et al., 2020)
*CB6*	lgG	Human	Competes with ACE2 for binding to RBD (Shi et al., 2020)
*B38 H4*	lgG	Human	Competes with ACE2 for binding to RBD (Dong et al., 2020; Jahanshahlu and Rezaei, 2020)
*B5*	lgG	Human	Binds to RBD but partially competes with ACE2 (Dong et al., 2020; Jahanshahlu and Rezaei, 2020)
*H2*	lgG	Human	Binds to RBD but displays no competition with ACE2 (Dong et al., 2020; Jahanshahlu and Rezaei, 2020)
*P2C-1F11*	lgG	Human	Disturbs the RBD-ACE2 interaction (Ju et al., 2020)
*P2B-2F6*			
*P2C-1A3*			
*rRBD-15*	lgG	Human	Prevents the interaction of RBD-ACE2 (Ho, 2020; Yu et al., 2020)
*311mAb–31B5*	lgG	Human	Blocks the binding of RBD-ACE2 (Chen X. et al., 2020)
*311mAb–32D4*			
*CC12.1*	lgG	Human	Targets the RBD-A epitope (Rogers et al., 2020)
*COVA1-18*	lgG	Human	Disturbs the RBD-ACE2 interaction (Brouwer et al., 2020)
*COVA2-15*			
*H11-D4*	lgG	Human	Disturbs the RBD-ACE2 interaction (Huo et al., 2020a)
*H11-H4*			
*COV2-2196*	lgG	Human	Completely competes with ACE2 for binding to RBD (Zost et al., 2020)
*COV2-2130*			
*2-15*	lgG	Human	Disturbs the RBD-ACE2 interaction (Yu et al., 2020)
*HA001*	lgG	Human	Disturbs the RBD-ACE2 interaction (Yi et al., 2020)
*II62*	lgG	Human	Disturbs the RBD-ACE2 interaction (Parray et al., 2020)
*n3088*	Nanobody	Human	Targets a cryptic epitope situated in RBD (Dong et al., 2020; Wu et al., 2020)
*n3130*			
*CV30*	lgG	Human	Prevents the S-ACE2 interaction (Seydoux et al., 2020)
*CV1/CV35*			

This signal-domain antibody 3F11 completely blocks the binding of RBD to ACE2 and shows neutralizing activity with an IC_50_ value of 0.4360 μg/mL in a SARS-CoV-2 virus neutralization assay. Moreover, in a SARS-CoV-2 pseudotyped virus entry assay, when sdAbs fuses with the human lgG1 crystallizable fragment (Fc) fragment, the recombinant 3F11 neutralizes against SARS-CoV-2 with an IC_50_ value of 0.0020 μg/mL (Yu et al., 2020).

BD-368-2 was isolated from 60 donors by using high-throughput single-cell RNA and VDJ sequencing of antigen-enriched B cells, which neutralizes the pseudotyped SARS-CoV-2 and authentic SARS-CoV-2 with an IC_50_ of 1.2 and 15 ng/mL, respectively. BD-368-2 targets the epitope which overlaps with the ACE2-RBD binding domain; therefore, BD-368-2 could be an effective NMAbs-targeting RBD (Cao et al., 2020).

A high-affinity mAb, IgG1 ab1, was rapidly identified from phage-displayed Fab, single-chain fragment variable region (scFV), and VH libraries. IgG1 ab1 shows neutralizing activity against authentic SARS-CoV-2 infection through preventing RBD to ACE2. The sequence of IgG1 ab1 has a relatively low number of mutations and no safety deficiencies; thus, lgG1 ab1 has become a potential therapeutic for SARS-CoV-2 infection (Li W. et al., 2020).

CB6, which was identified through human B cells of convalescent donors, prevents the binding of RBD to its receptor ACE2 by steric hindrance and competition with interface residues. CB6 exhibits neutralizing activity against pseudovirus SARS-CoV-2 with ND_50_ values of 0.036 μg/ml (Shi et al., 2020).

Four antibodies, B38, H4, H2, and H4, which are bound to the epitopes of RBD, are identified from the peripheral blood of COVID-19 convalescent donors. H4 suppresses SARS-CoV-2 infection with an IC_50_ value of 0.896 μg/mL, while B38 has a higher potency to inhibit SARS-CoV-2 virus infection with an IC_50_ value of 0.177 μg/mL. The two antibodies act on different domains, so there is no competition between B38 and H4, which enables them to be used in combination for targeting the virus to prevent immune escape in clinics (Dong et al., 2020; Jahanshahlu and Rezaei, 2020).

P2C-1F11, P2C-1A3, and P2B-2F6 are screened from the plasma of convalescing patients. The P2B-2F6 Fab competes with the S protein to block the ACE2–RBD binding. P2C-1F11, P2C-1A3, and P2B-2F6 are strong inhibitors of authentic SARS-CoV-2, with IC_50_ values of 0.03, 0.28, and 0.41 μg/mL, respectively (Ju et al., 2020). rRBD-15 is identified through a synthetic human Fab antibody library and exhibits the neutralizing activity against SARS-CoV-2 pseudovirus infection with an IC_50_ value of 12.2 nM (Ho, 2020; Yu et al., 2020). 311mab-32D4 and 311mab-31B5 could prevent the binding of RBD to its receptor hACE2 with IC_50_ values of 0.0450 and 0.0332 μg/mL, respectively. Moreover, 311mab–32D4 and 311mab–31B5 show the neutralizing activity against SARS-CoV-2 with IC_50_ values of 0.0698 and 0.0338 μg/mL in a SARS-CoV-2 pseudotyped virus entry assay, respectively (Chen X. et al., 2020).

Identified from B cells of convalescent donors by a high-throughput rapid system, CC12.1 shows neutralizing activity against SARS-CoV-2 with an IC_50_ value of 0.019 μg/mL in a SARS-CoV-2 pseudotyped virus entry assay and with an IC_50_ value of 0.022 μg/mL against authentic SARS-CoV-2 infection. In addition, CC6.30 and CC6.29 show neutralizing activity against SARS-CoV-2 with IC_50_ values of 1 ng/mL and 2ng/mL in a SARS-CoV-2 pseudotyped virus entry assay, respectively (Rogers et al., 2020).

Another two antibodies, COVA2-15 and COVA1-18, were obtained from B cells of convalescent donors. These antibodies have shown neutralizing activity against SARS-CoV-2 pseudovirus infection with an IC_50_ value of 8 ng/mL for both and live neutralization SARS-CoV-2 infection with IC_50_ values of 7 and 9 ng/mL (Brouwer et al., 2020).

H11-H4 and H11-D4 target epitopes that are closely adjacent to and are slightly overlapping with the ACE2-binding domain to block RBD–ACE2. Furthermore, when nanobodies H11-D4 and H11-H4 are fused with lgG Fc, they also show the neutralizing activity against SARS-CoV-2. Nanobodies H11-H4 and H11-D4 bind with RBD with KD of 12 and 39 nM, respectively. Studies have shown that H11-D4 and H11-H4 have the additive neutralizing activity against SARS-CoV-2 with the CR3022 antibody because it recognizes a different binding site. Such a strategy is a good way to reduce the possibility of the virus escape by mutation (Huo et al., 2020a).

COV2-2196 and COV2-2130, identified from the lymphocytes of convalescent donors, neutralize the SARS-CoV-2 pseudovirus infection by binding to different sites on RBD and completely prevent the interaction with hACE2. Therefore, both COV2-2196 and COV2-2130 could bind to the S protein simultaneously and exhibit a synergistic neutralizing effect on SARS-CoV-2 infection. The use of COV2-2196 and COV2-2130 alone or in combination is promising for the treatment or prevention of SARS-CoV-2 (Zost et al., 2020).

Yu et al. identified 61 NMAbs from peripheral blood mononuclear cells (PBMCs) of five severe SARS-CoV-2 donors, among which nine NMAbs have been identified against the authentic SARS-CoV-2 infection. The most potent NMAb 2-15 could neutralize pseudotyped and authentic SARS-CoV-2 infection through competitively binding RBD with ACE2, with IC_50_ of 5 and 0.7 ng/mL, respectively (Yu et al., 2020).

A recently developed SARS-CoV-2 receptor-blocking human antibody, HA001, was found to interact with new binding sites (A475 and F486 in RBD) for neutralizing antibodies in SARS-CoV-2 but could not recognize SARS-CoV. The study indicates that there are different key epitopes between SARS-CoV and SARS-CoV-2, which provide insights to develop more specific antibody drugs and prophylactic vaccine (Yi et al., 2020).

Using an scFv phage display library, II62, a novel scFv was identified. II62 targets a region that is adjacent and closely overlapping with the binding domain of ACE2 on the RBD. Hilal et al. found II62–scFv and its two other formats, scFv-Fc and IgG1, that target the RBD and interrupt the ACE2–RBD binding. The inhibition of RBD to its receptor ACE2 is dependent on the concentration (starting from 5 to 0.008μM). Upon tested up to 100 μg/mL against authentic SARS-CoV-2 virus, II62 does not exhibit a significant reduction in the neutralizing potential. The possible explanation is that it is only accessible in the open post-fusion conformation of the S protein. S309 and S310 recognize post-fusion conformation of SARS-CoV-2 S protein, and CR3022 exhibits non-neutralizing behavior against SARS-CoV-2. Non-NAb plays an essential role in the blockage of cell surface infection *in vivo* without perturbing virus entry, which could be useful for improving antibody-based therapeutics and diagnostics for COVID-19 (Parray et al., 2020).

A nanobody called n3088 was isolated from a full human sdAb library and exhibits neutralizing activity against SARS-CoV-2 by targeting a cryptic epitope located in RBD of SARS-CoV-2 (Dong et al., 2020; Wu et al., 2020).

Upon isolating B cells specific to the SARS-CoV-2 S protein from patients infected with SARS-CoV-2 after 21 days of clinical disease onset, CV30 and CV1/CV35 exhibit neutralizing activity against SARS-CoV-2. Moreover, CV30 binds RBD in a manner that prevents the binding of ACE2 to RBD. CV1/CV35 binds to a domain distant from the RBD and has less neutralizing activity than CV30 (Seydoux et al., 2020).

## Vaccine Based on the RBD Region

**FIGURE 2:**
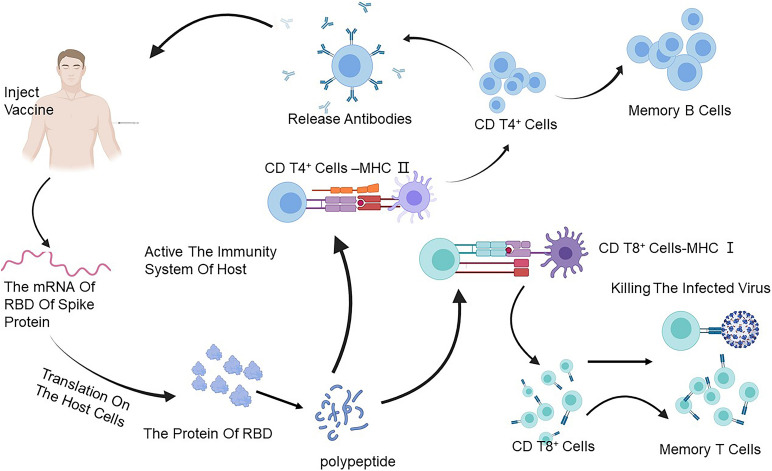
The immune response caused through a vaccine. The mRNA of the RBD of the S protein of SARS-CoV-2 can translate on the ribosome of the host, and then the foreign protein of RBD reduces to a polypeptide to stimulate the immune system and is caught by the antigen-presenting cells (APCs) which can process the vaccine antigen and present it to the CD4 T^+^ cells and CD8 T^+^ cells. It acquires the ability to treat SARS-CoV-2 by activating B cells to produce antibodies and T cells to attack the infected cells. Created by BioRender.com. SARS-CoV-2, severe acute respiratory syndrome coronavirus-2; RBD, receptor-binding domain.

To date, over 150 SARS-CoV-2 vaccines are under development all over the world. Most recently, RBD-based SARS-CoV-2 vaccine candidates could induce a strong RBD-based neutralizing antibody response in some animals after just one dose of injection (Figure 2) (Yang J. et al., 2020). Herein, we summarize the development of vaccines that could induce antibodies specific to RBD (Tables 3, 4).

**TABLE 3:** The clinical stage of vaccines targeting the RBD of SARS-CoV-2.

Vaccine	Developer	Clinical stage number of doses	Timing of doses
*AdimrSC-2f*	Adimmune Corporation	Phase 1 clinical trials	N/A
*Unnamed Recombinant Vaccine*	West China Hospital	phase 2 clinical trials	0, 21 days and 0, 14, 28 days (Yang J. et al., 2020)
*ZF2001*	Anhui zhifei longcom Biologic Pharmacy Co., Ltd	Phase 3 clinical trials	0, 30, and 60 days (Yang S. et al., 2020)
*BNT162b1*	BioNTech and Pfizers	Phase 1/2 clinical trials	0 and 28 days (Sahin et al., 2020)
*ARCoV*	Chinese Academy of Military Medical Sciences	Phase 1 clinical trials	N/A (Zhang et al., 2020)

**TABLE 4:** The clinical stage of monoclonal antibodies targeting the RBD of SARS-CoV-2.

Vaccine	Developer	Clinical stage number of doses	Trial ID	Monoclonal antibodies
*VIR-7831*	Vir Biotechnology	Phase2/3 clinical trials	NCT04545060	S309 (Tuccori et al., 2020)
*AZD7442*	AstraZeneca	phase 3 clinical trials	NCT04625972	COV2-2196 COV2-2130
*LY-CoV555*	AbCellera/Eli Lilly and Company	Phase 3 clinical trials	NCT04497987	A recombinant, fully human neutralizing IgG1 mAb (Tuccori et al., 2020)
*LY-CoV016*(JS016)	Junshi Biosciences/Eli Lilly and Company	Phase 2 clinical trials	NCT04427501	A recombinant fully human neutralizing monoclonal antibody
*CT-P59*	Celltrion	Phase 3 clinical trials	NCT04525079	A fully human neutralizing monoclonal antibody
*REGN-COV2*	Regeneron Pharmaceuticals	Phase 3 clinical trials	NCT04452318	REGN10987 REGN10933 (Baum et al., 2020b; Hansen et al., 2020)

Lu group developed a recombinant subunit vaccine candidate that contains the RBD of SARS-CoV-2 and the Fc fragment of human lgG, which can extend the half-life of the immunogen and enhance the responses of the long-term neutralizing antibody (Kontermann, 2011). It is a benefit for therapeutic and economic reasons to reduce the number of usage and dosage by the extension of the half-life. Recently, many mutations in SARS-CoV-2 S protein have emerged; thus, people are worried about if the current vaccines could also be helpful when those mutants exist in SARS-CoV-2. To evaluate the neutralizing activity of the RBD-Fc-based SARS-CoV-2 vaccine candidates, Liu et al. constructed eight RBD mutants of SARS-CoV-2 PSV with detectable infectivity. The results show that these natural mutations in RBD of SARS-CoV-2 PSV did not influence the neutralizing activity of the RBD-Fc-induced antisera. Moreover, the antibody induced by the RBD-Fc-specific SARS-CoV-2 vaccine could cross-neutralize the infection of a bat pseudotyped SARS-CoV WIV1 as well as the pseudotyped SARS-CoV (Liu et al., 2020).

AdimrSC-2f (NCT04522089) is a protein subunit vaccine developed by Adimmune Corporation, which is now under phase 1 clinical trials in Taiwan (clinicaltrials.gov). AdimrSC-2f can be used to immunize an individual, with or without aluminum content as an adjuvant, to bolster an immune response that could protect them from infection with the native virus.

The recombinant vaccine (NCT04640402), developed by West China Hospital, containing residues 319–545 of the RBD of the S protein, has been ongoing in phase 2 clinical trials in China. This protein subunit vaccine is expressed in Sf9 insect cells. Individuals can be immunized and generate a specific antibody against the S-RBD protein of SARS-CoV-2, which could protect them from the infection with the native virus (Yang J. et al., 2020).

ZF2001 (NCT04646590) is a recombinant protein vaccine that uses a dimeric fragment of RBD as the antigen to trigger a protective immune response. The vaccine is well tolerated and immunogenic, which is in phase 3 clinical trials in China (Yang S. et al., 2020).

A lipid nanoparticle–formulated, nucleoside-modified mRNA vaccine, BNT162b1 (NCT04368728), encodes the trimerized RBD of the S protein of SARS-CoV-2 (Sahin et al., 2020). The RBD antigen encoded through BNT162b1 is fused to a T4 fibrin–derived fold by trimerization to enhance its immunogenicity through a multivalent display (Tai et al., 2016). Once vaccinated, host cells uptake the mRNA and then generate the protein which touches off the immune response against infection with SARS-CoV-2. On September 30, 2020, the BNT162b1 vaccine of BioNTech and Pfizer has entered into Phase 1/2 clinical trials, and the results show that the immunized individuals elicit a strong SARS-CoV-2-specific antibody and CD4^+^ and CD8^+^ immune responses that suggest multiple mechanisms with the potential to protect against SARS-CoV-2. However, the storage condition needs ultra-low temperature of −70°C in a freezer, which makes it difficult to transport and store, so it is difficult to reach low-income countries due to high cost.

ARCoV, a lipid nanoparticle–encapsulated mRNA vaccine, encodes the RBD of SARS-CoV-2, which can induce strong antibodies and arouse T-cell responses against multiple epidemic SARS-CoV-2 strains in some animals. Importantly, compared with other mRNA vaccines, it could be kept at room temperature for at least 7 days. The vaccine has been tested in phase 1 clinical trials (Zhang et al., 2020).

The two NAbs, REGN10987 and REGN10933 (NCT04519437), bind two distant regions of RBD, where the epitope of REGN10987 is on the side of the RBD, while the epitope of REGN10933 locates away from that of REGN10987, which is an advantage to avoid generating escape mutants. The variant of D614G has a pandemic in the world, but the neutralizing potency of REGN10933 and REGN10987 is not affected in the presence of this variant (Baum et al., 2020a; Yurkovetskiy et al., 2020). The combination of antibodies has been tested in phase 3 clinical trials (Baum et al., 2020b; Hansen et al., 2020).

VIR-7831 (GSK 4182136), which has entered phase 2/3 clinical trials, is a fully human mAb, based on the S309 which is identified from a SARS survivor, that targets the RBD of SARS-CoV-2 and is developed by Vir Biotechnology and GSK.

AZD7442 is composed of two human mAbs, COV2-2196 and COV2-2130, which are isolated from the lymphocytes of convalescent patients, that exhibit neutralizing activity against SARS-CoV-2 by disturbing the binding of ACE2–RBD. Furthermore, COV2-2196 and COV2-2130 exhibit strong neutralizing activity against some variations, such as E484K, N501Y, and D614G. AZD7442 is in phase 3 clinical trials.

LY-CoV555 is a neutralizing antibody identified from a SARS-CoV-2 survivor. LY-CoV555 has a high-affinity binding with RBD, thus exhibiting neutralizing activity against SARS-CoV-2. Recently, some studies showed that the combination of two antibodies, LY-CoV555 and LY-CoV016, have strong neutralizing activity against SARS-CoV-2 and can reduce hospitalizations and death for patients with early-stage COVID-19.

CT-P59 developed by Celltrion has entered phase 3 clinical trials. It could improve the recovery times of patients with COVID-19 and reduce progression rates of SARS-CoV-2.

## Conclusion

SARS-CoV-2 has led to a serious ongoing global disaster and imposes a great threat to the economy and health management system all over the world. Hundred million people have been confirmed of the SARS-CoV-2 infection, and over 2 million people have died till February 2, 2021. Even though several positive efficacy results have been reported on the vaccine candidates, SARS-CoV-2 vaccines developed so far cannot meet the global demand. Vaccine candidates against SARS-CoV-2 have different compositions, from traditional whole-pathogen vaccines, live-attenuated vaccines, and inactivated vaccines to various new-generation recombinant protein vaccines and vector-based vaccines. Instead of the whole virus, the new generation vaccines only contain a specific antigen or antigen from the pathogen that makes the vaccine safer than the traditional vaccines. The vaccines we discuss are mainly recombinant protein vaccines and vector-based vaccines. Because the recombinant protein vaccines use a protein fragment of RBD as the antigen, this type of vaccine has lower immunoreactivity than the whole-pathogen vaccines, but the immunogenicity of the recombinant protein vaccines is weaker than others which need multiple vaccine dosages or adjuvants, and it is easy to store and transport. The disadvantage of the recombinant subunit vaccine is that it is difficult to design a good structure of a recombinant protein and the expression system. Furthermore, this type of vaccine may need adjuvants to enhance the immunogenicity of the vaccines. As a nucleoside-modified mRNA, which is the advent of a new era in vaccinology, there are many advantages of this type of vaccine; for instance, they pose no risk of genomic integration, are easy to design, and can be manufactured rapidly. However, the mRNA vaccine need to be encapsulated; otherwise, it becomes unstable under physiological conditions, and the half-life of mRNA is short; thus, there may be many problems or side effects beyond our expectations (Koirala et al., 2020; Wang J. et al., 2020), because the technology is too new, and there is no successful precedent, so most of the countries cannot carry out industrial production, and it may be difficult to popularize in low-income countries because of the high price. What is worse, SARS-CoV-2 is undergoing mutation, which makes it more difficult to explore vaccines. Among them, D164G is of particular note because that itself and its combination with other mutations have made the virus more infectious. Moreover, some variants, such as V483A, N234Q, Y508H, A475V, F490L, L452R, and N439tK, also have reduced sensitivity to neutralizing mAbs (Li Q. et al., 2020). Therefore, it is urgent to develop broad-spectrum vaccines, neutralizing antibodies, and other therapeutic tools to fight against SARS-CoV-2.

## Author Contributions

LM wrote the manuscript. QS revised the manuscript. Both authors contributed to the article and approved the submitted version.

## Conflict of Interest

The authors declare that the research was conducted in the absence of any commercial or financial relationships that could be construed as a potential conflict of interest.
